# Management of Perforating Idiopathic Internal Root Resorption 

**DOI:** 10.22037/iej.2017.50

**Published:** 2017

**Authors:** Dalia Abdullah, Farah Eziana Hussein, Huwaina Abd Ghani

**Affiliations:** a *Department of Operative Dentistry, Faculty of Dentistry UKM, Jalan Raja Muda Abdul Aziz, 50300 Kuala Lumpur, Malaysia;*; b *School of Dental Science, USM Health Campus, Kubang Kerian, 16150 Kota Bharu, Kelantan, Malaysia*

**Keywords:** Cone-Beam Computed Tomography, Idiopathic, Internal Root Resorption, Mineral Trioxide Aggregate

## Abstract

This case report describes the endodontic treatment of an idiopathic perforated internal root resorption. A 24-year-old male Malay patient presented with internal root resorption of two of his anterior teeth. The medical history was non-contributory and he had no history of traumatic injury or orthodontic treatment. Cone-beam computed tomography (CBCT) determined the nature, location and severity of the resorptive lesion. Non-surgical root canal treatment of tooth #22 and combined non-surgical and surgical approach for tooth #11 were carried out using mineral trioxide aggregate (MTA) as the filling material. The clinical and radiographic examination three years after completion of treatment revealed evidences of periapical healing. The appropriate diagnosis and the treatment of internal root resorption allowed good healing of these lesions and maintained the tooth in function for as long as possible.

## Introduction

Internal root resorption (IRR) is an inflammatory condition that results in progressive destruction of intra radicular dentin along the middle and apical thirds of the canal walls due to osteoclastic action [1, 2]. It is relatively rare in occurrence, and the aetiology and pathogenesis of the lesion have not been completely understood in comparison with external root resorption [3].

IRR could be asymptomatic and may present on routine radiographic examinations [1]. Patient might experience symptoms of pulpitis at the initial stage [1]. At a later stage, the root canal system may become necrotic and patient might eventually develop symptoms of periradicular periodontitis [1, 4].

Diagnostic accuracy of internal root resorption based on conventional radiographic examination is limited by the fact that the produced images only provide a two-dimensional representation of three-dimensional object. Radiographic imaging is unable to reveal the location and nature of the resorptive defect as well as the thickness of the remaining root canal dentine [5]. Furthermore, the anatomic structures might be superimposed and image might be distorted [6]. The introduction of cone-beam computed tomography (CBCT) in endodontics has enhanced radiographic diagnosis and management of resorptive lesions [7, 8]. CBCT provides information such as the size, shape and nature of the lesion including root perforations [5]. 

This case report describes the successful management of an idiopathic perforated IRR associated with two anterior teeth.

## Case Report

A healthy 24-year-old male Malay patient was referred by his dentist to the endodontic specialist clinic in 2011 for the management of teeth #11 and 22. The patient reported an acute episode of throbbing pain approximately two weeks prior to his referral. He was taking a course of antibiotic prescribed by the dentist. The medical history was non-contributory. He had no history of traumatic injury or orthodontic treatment

**Figure 1:**
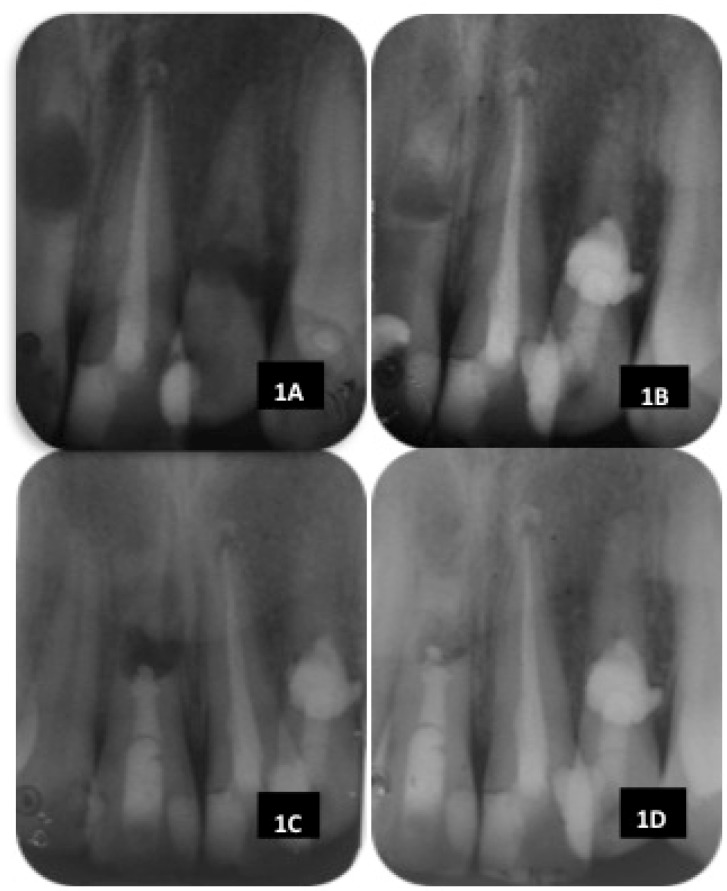
A) Preoperative radiograph; B) One-year review of tooth 22; C) Postoperative of tooth 11 filled with MTA; D) Nine months review post-surgery of tooth 11

In the clinical examination, teeth #11 and 22 had discoloured mesial composite restorations with intact margins. The probing depth was within normal limit for both teeth. Tooth #11 was tender on palpation and percussion elicited a different sound. The tooth was not mobile. Soft swelling of buccal tissues was noted at apical area of tooth #11 and it was also tender to palpation. A periapical radiographic examination revealed a large, well demarcated oval radiolucency at the middle third of root canal of tooth #11 with the remaining small portion of the apex (Figure 1A). 

Tooth #22, was not tender to percussion and palpation nor mobile. Pulp sensibility test (Elements Diagnostic Unit, SybronEndo, Orange, California) was performed and gave a positive reading at 20. Radiographic examination revealed a radiolucent area at the cervical third of the root canal and the canal could not be followed through the lesion (Figure 1A). The defect could not be probed through gingival sulcus. A diagnosis of idiopathic internal root resorption of tooth #11 and 22 was made. An orthopantomogram (OPG) radiography was also taken to assess all the other teeth and no other resorptive lesion was noted.

**Figure 2:**
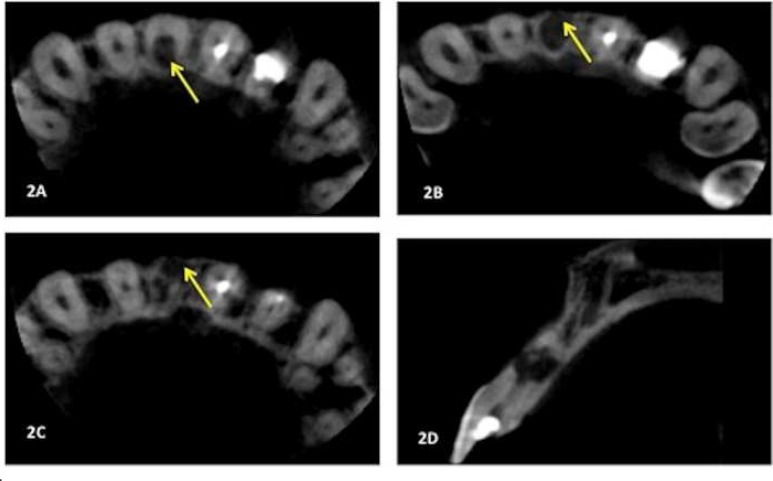
A) Axial slice demonstrating perforation of palatal root surface; B) Axial slice demonstrating perforation of buccal root surface; C) Axial slice showing a buccal bone plate perforation; D) Sagittal slice showing the location and extension of the resorptive lesion

To assess the nature, location and severity of the resorptive lesion, limited CBCT 3D (Kodak 9000D, Carestream, Health Inc, Kodak Dental Systems, Marne-la-Valee, France) of the region associated with tooth #11 was taken after verbal consent obtained. Following analysis of the axial, sagittal and coronal slices, it was evident that the internal root resorption of tooth #11 was extensive and had perforated at the middle third of canal in the palatal (Figure 2A) and buccal aspects (Figure 2B) with buccal bone plate penetration at middle third of the root (Figure 2C). Extent of the resorption could be seen from the sagittal view (Figure 2D).

The patient was informed of the clinical findings, and various treatment options were discussed. The potential technical difficulties, in particular, the problem of effective sealing of the resorptive defect and the poor prognosis of the endodontic treatment for both teeth were explained to the patient. The agreed treatment plan was; *1)* emergency treatment of tooth #11 as it was symptomatic, *2)* non-surgical root canal treatment of tooth #22 to arrest the progressive resorption and *3)* combined non-surgical and surgical approach for tooth #11. 

At the first visit, the root canal of tooth #11 was accessed and there was pus and blood present in the canal. Canal was cleaned and dressed with calcium hydroxide and access cavity was filled with temporary cement (Kalzinol, De Trey Dentspiy).

Treatment was then carried out for tooth #22. The root canal was accessed under surgical microscope (OPMI Pico Zeiss Dental Microscope, Germany) and there were lots of tissue in the resorptive cavity and excessive bleeding present in the canal. It was not possible to negotiate the canal beyond the resorption even after two visits. Mineral trioxide aggregate (MTA) (ProRoot MTA; Dentsply Tulsa Dental, Tulsa Oklahoma, USA) was placed in the resorptive cavity up to the orifice of the canal.

**Figure 3:**
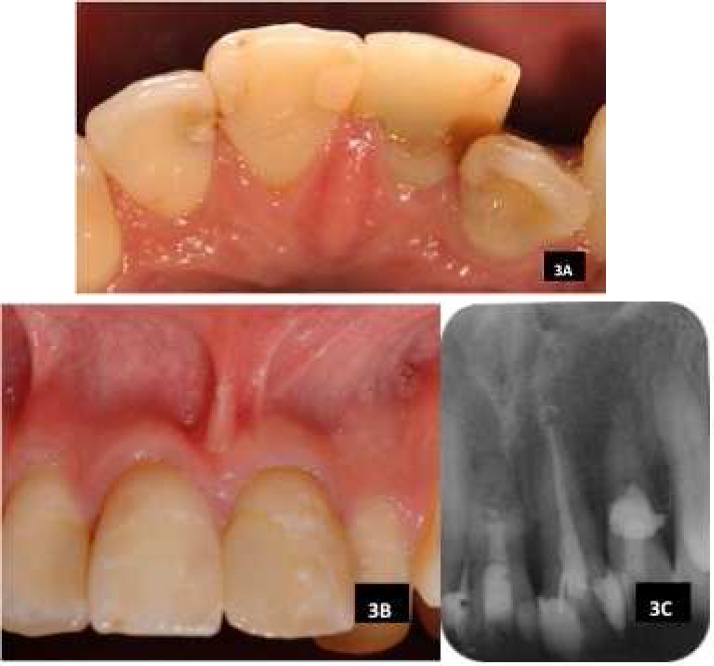
A) Palatal view of tooth 11 and 22; B) Buccal view of tooth 11 and tooth 22; C) Periapical radiograph three years after treatment completion

Patient was scheduled for the management of tooth #11. However, he was out of the city and came back to see us a year later in 2012. Upon review, the tooth #22 was asymptomatic and periapical radiography showed absence of periradicular radiolucency (Figure 1B). Root canal of tooth #11 was accessed and perforation was detected under the microscope. With the aid of CBCT scan, an accurate measurement of the perforation level could be obtained, which was at the length of 15 mm. The 27-gauge irrigating needle was pre-measured to ensure that there was no inadvertent extrusion of irrigant into the periradicular tissue. The apex was large with no apical stop. MTA was placed at the apex up to the coronal part of the canal and access cavity was filled with Kalzinol (Figure 1C). At the same visit, a buccal flap was raised and the granulation tissue was removed during the surgery. Attempting for removal of the apical end of the root was not successful. Flap was then repositioned and sutured. Review was done two weeks after the surgery, the patient was pain-free, and was able to eat and brush. Clinical examination showed the surgical incision site was healing well where there was a pink fibrous gingival mucosa observed. At nine months review after surgery of tooth #11, both teeth were asymptomatic with healthy clinical signs. Periapical radiograph (Figure 1D) showed that the radiolucent area had reduced in size. 

The patient was reviewed again recently three years after treatment completion. Patient was asymptomatic and clinically, the teeth and the surrounding tissues were healthy (Figure 3A and 3B). The periapical radiograph appeared normal with re-establishment of bony structure around the roots (Figure 3C).

## Discussion

Management of IRR is an endodontic challenge especially if the resorptive lesion is extensive and perforating. A correct diagnosis is important as the management of IRR is different from ERR. Once the diagnosis and prognosis of IRR have been established, root canal treatment is the treatment of choice [2]. The rate of resorption may be rapid or slow and spontaneous repair is extremely rare, thus the *wait and see* approach is not appropriate [9]. The aim of root canal treatment is to arrest the cellular activity responsible for the resorptive lesion by removing all the causative agents [10], disinfect and obturate the root canal system [1]. However, the complex irregularities of the root canal system and the inaccessibility of IRR defect provide technical difficulties for thorough cleaning and obturation of the root canal [11]. Sodium hypochlorite and calcium hydroxide used in the chemomechanical debridement is very important to disinfect the root canal system. Failure to remove the bacteria and organic debris in those areas may jeopardize the long-term success of the endodontic treatment [12]. 

Radiographically, internal resorption may be described as symmetrical or eccentric lesion with sharp, smooth and clearly defined margin, with a uniform density of radiolucency, and the outline of pulp chamber or root canal could not be followed through the lesion [13]. In contrast, lesions caused by ERR may be asymmetrical and have ill-defined borders, with radio density variations in the body of the lesion [14]. The canal wall should be traceable through ERR lesion because ERR is superimposed over the root canal. Parallax method is recommended for distinguishing internal and external resorptive defects [13]. A second radiograph taken from a different mesio-distal angle would alter the relationship of the defect to the root canal but not in the case of IRR [6]. 

Several case reports and studies have confirmed the usefulness of CBCT in diagnosing and managing resorptive lesions [6, 14]. Although diagnosing IRR is primarily made based on periapical radiograph, certain informations are lacking. In this present case, the complexity of the resorptive lesion with presence of perforation was clearly visible in CBCT images. This valuable information was shown to be extremely useful in the diagnosis, making a proper treatment plan and management during treatment was being carried out. In the case of perforating IRR of tooth #11, information obtained from CBCT scans means that greater care could be taken during root canal irrigation. As the exact location of the perforation had been determined preoperatively, the position and orientation of the side-venting irrigation needle tip could be controlled to minimize the risk of extrusion of sodium hypochlorite solution. 

Information obtained from CBCT scan also influenced the method of root canal filling used. Internal root resorption defects can be difficult to obturate adequately. In this case, there would be a problem of significant extrusion of root filling material through the perforation if flowable gutta percha was used. MTA was chosen instead because of its superior sealing properties, bacteriostatic effects, biocompatibility and its ability to set in presence of blood [15]. Surgical endodontic treatment was carried out for tooth #11 to remove the resorptive tissues and the apical portion. The use of MTA as orthograde filling eliminated the need to place a root-end filling at the time of surgery. 

The prognosis of conventional root canal treatment for non-perforated IRR is high which was in accordance to previously reported study [9]. However, the formation of calcific barrier for the treatment of perforating resorptive defect is poor [9]. In this case, although the resorption of tooth #11 was extensive and perforation had also involved the buccal bone plate evaluation showed complete healing of the tissues.

## Conclusion

This case report was intended to share information on management of idiopathic internal root resorption, the use of CBCT in decision-making process and MTA as the material of choice to treat root perforation. Diagnosing the types of root resorption lesion is a challenge as the clinical and radiographic presentation might be similar. CBCT has been very beneficial in the assessment and management of perforating internal root resorption. In internal root resorption, when endodontic treatment is adequate, lesion can be halted and evidence of bony healing can be seen radiographically.
